# Diagnostic value of magnetic resonance imaging and the outcomes of medical therapy in histologically confirmed diabetes-related foot osteomyelitis

**DOI:** 10.5194/jbji-10-411-2025

**Published:** 2025-10-29

**Authors:** Abhay Mishra, Paige Lyell, David M. Bamberger

**Affiliations:** 1 Division of Infectious Diseases, Department of Medicine, University of Missouri-Kansas City School of Medicine and University Health/Truman Medical Center, Kansas City, MO, USA

## Abstract

**Background**: The preferred imaging modality in diabetes-related foot osteomyelitis is magnetic resonance imaging; however the test characteristics of magnetic resonance imaging have not been well evaluated in histologically confirmed disease. Although there is reasonably strong evidence of the effectiveness of medical therapy in the treatment of diabetes-related foot osteomyelitis, there is limited evidence in histologically confirmed cases. **Methods**: A retrospective review of episodes in which diabetes-related foot osteomyelitis was suspected was performed among patients who had both magnetic resonance imaging and bone histology. Magnetic resonance images were reviewed by a musculoskeletal trained radiologist using guideline criteria. Cases were subsequently followed for need for amputation at 1 year. **Results**: Among 84 episodes, the sensitivity of magnetic resonance imaging in histologically confirmed diabetes-related foot osteomyelitis was 96 %, specificity 38.2 %, positive predictive value 69.6 %, negative predictive value 86.7 %, positive likelihood ratio 1.15, and negative likelihood ratio 0.1. Of 15 cases in which the histology specimen revealed osteomyelitis from a diagnostic biopsy, 10 responded to medical management without the need for an amputation. **Conclusions**: Magnetic resonance imaging is useful excluding diabetes-related foot osteomyelitis but is of limited diagnostic value in confirming its presence. Most cases of histologically confirmed diabetes-related foot osteomyelitis responded to medical management without the need for amputation.

## 1 Introduction

Diabetes-related foot osteomyelitis (DFO) is a common entity with resultant frequent hospitalizations, morbidity, and amputations. The diagnosis of osteomyelitis in those with diabetes-related foot infection therefore is critical to provide effective therapy. The gold standard in the diagnosis includes the demonstration of histologic evidence of bone infection (Berendt et al., 2008; Lipsky et al., 2012). The use of bone biopsies, however, has not been clearly demonstrated to enhance outcomes. The preferred radiographic technique in the diagnosis of diabetes-related foot osteomyelitis (DFO) that has been best studied is magnetic resonance (MR) imaging. The reported sensitivity and specificity of MR imaging in the diagnosis of DFO in systematic reviews and meta-analyses are 93 %–96 % and 75 %–82 %, respectively (Lauri et al., 2017; Llewellyn et al., 2020). However, most reports evaluating the role of MR imaging were not compared to a diagnostic gold standard of histology. Further, previous studies demonstrating the role of medical therapy in the management of DFO were not based on those only with histologically proven disease.

The primary purpose of our study was to evaluate the sensitivity, specificity, likelihood ratios, and predictive values of MR imaging in the diagnosis of histologically confirmed DFO. We further evaluated the test characteristics in those patients in whom histology was obtained via a surgical amputation compared to a diagnostic bone biopsy. As a secondary outcome, we provided outcomes on those who had medical treatment only for those with biopsy-proven DFO and provided additional information on the risk factors for successful outcome.

## 2 Methods

Cases were enrolled retrospectively between 1 June 2009 and 31 July 2019 at a single academic safety-net institution among patients who had a diabetes-related foot infection who had both MR imaging and a subsequent specimen for histologic exam via either a biopsy or surgical resection within 60 d. Cases were excluded if there was foot tumor, recent surgery, or recent injury. Cases were included from the same patient if a subsequent infection involved a different anatomic site. All radiology reports were reviewed for the presence or absence of findings of osteomyelitis. Cases in which diagnostic uncertainty was implied by the radiology report were re-reviewed for a final determination by a board-certified musculoskeletal radiologist utilizing Society of Skeletal Radiology practice guidelines (Alaia et al., 2021) who was blinded to results of the histology. A positive result was then there was marrow edema and confluent T1 hypointense signal in the bone with an adjacent infection/ulcer. A negative result was when there was normal marrow signal. A suspicious result was when there was an overlying ulcer with marrow edema but no confluent T1 signal. An indeterminate result was recorded if there were technical issues such as movement, poor fat suppression, fractures, or bone infarcts. When a diagnostic biopsy of bone was performed in obtaining the histologic sample, the podiatrist or surgeon utilized an 11G Jamshidi biopsy needle or a bone rongeur and did not necessarily attempt to avoid going through an overlying ulcer if present. Cases were followed subsequently to determine the need for amputation at 1 year.

## 3 Results

### 3.1 Patient characteristics and diagnostic value of MR imaging compared to histology

Among 63 patients, 84 encountered cases were enrolled. In total, 1 patient had four encounters, 3 patients had three encounters, 12 patients had two encounters, and 47 patients had a solitary encounter. The clinical characteristics of each patient encounter are shown in Table 1.

**Table 1:** Clinical characteristics of encountered patients.

Age (years)^*^	56 (49–60)
Male/female gender (number)	66/18
Length of ulceration or symptoms prior to MR (days)^*^	77 (28–187)
Glycosylated hemoglobin^*^	8.5 (7.0–11.5)
Estimated glomerular filtration rate^*^	76 (54–90)
Site of infection (number)	
Toe only	38
Metatarsal ± distal toe	37
Cuboid ± distal metatarsal	3
Calcaneus	6

^*^ Median (interquartile range).

In the final radiographic interpretation, 63 of the MR images were interpreted as positive for osteomyelitis, 10 as negative, 6 as suspicious, and 5 as indeterminate (Table 2). The median time from MR imaging to obtaining the bone sample for histology was 3 d; 81 % of patients had the procedure done within 1 week and 90 % within 2 weeks. All but one patient had the procedure performed within 25 d. In total, 41 patients had the sampling of bone done for histology during the time of a therapeutic amputation and 43 as part of a diagnostic bone biopsy. In 50 patient encounters, the diagnosis of osteomyelitis was confirmed histologically, including in 35 of 41 in which the sample was obtained by amputation and in 15 of 43 in which the sample was obtained by diagnostic biopsy.

**Table 2:** Results of MR imaging and histology.

		Encounters	Histology +	Histology -
MR scan total group	positive	63	47	16
	suspicious	6	1	5
	negative	10	1	9
	indeterminate	5	1	4
	total	84	50	34
MR scan in those with amputation	positive	36	32	4
	suspicious	2	1	1
	negative	2	1	1
	indeterminate	1	1	0
	total	41	35	6
MR scan in those with diagnostic biopsy	positive	27	15	12
	suspicious	4	0	4
	negative	8	0	8
	indeterminate	4	0	4
	total	43	15	28

The results of the test characteristics of MR scanning in the diagnosis of osteomyelitis are shown in Tables 2 and 3. In Table 3, we calculated disease prevalence, sensitivity, specificity, likelihood ratios, and predictive values first in the total group by deeming those with suspicious scans as positive and those with indeterminate scans as negative and then again by excluding those with MR scans read as suspicious and indeterminate. We also calculated the test characteristics in the entire group and then again in the group that had a surgical therapeutic amputation and in those who had a diagnostic biopsy. For all groups, the sensitivity of MR scanning was high but the specificity poor. Among the 43 patients who had a diagnostic biopsy, 27 had positive MR findings, but only 15 of these had histologic evidence of osteomyelitis, with a resultant positive predictive value of 55.6 % (95 % CI 37 %–73 %) and positive likelihood ratio of only 0.175. However, MR scanning was useful in excluding osteomyelitis in this group, with no false negative scans, a negative predictive value of 100 % (95 % CI 74 %–100 %), and a negative likelihood ratio of 0.00.

**Table 3:** Sensitivity, specificity, predictive values, and likelihood ratios of MR imaging compared to histology.

	N	Prevalence	Sensitivity	Specificity	PPV	NPV	LR +	LR -	Post test probability positive test
Total group^a^	84	59.5 %	96.0 (86–99)	38.2 (23–56)	69.6 (57–80)	86.7 (60–96)	1.55	0.1	69 %
Amputation group^a^	41	85.4 %	97.1 (85–99)	28.6 (8–58)	86.8 (73–94)	66.7 (20–94)	1.36	0.10	87 %
Diagnostic biopsy group^a^	43	34.9 %	100.0 (78–100)	42.9 (25–62)	48.4 (31–66)	100.0 (74–100)	1.75	0.00	48 %
Total group^b^	73	65.8 %	97.9 (89–100)	36.0 (20–55)	74.6 (62–84)	90.0 (59–99)	1.53	0.06	75 %
Amputation group^b^	38	86.8 %	97.0 (84–99)	20.0 (1–62)	88.9 (74–96)	50.0 (9–91)	1.21	0.15	89 %
Diagnostic biopsy group^b^	35	42.9 %	100.0 (78–100)	40.0 (22–61)	55.6 (37–73)	100.0 (74–100)	1.67	0.00	56 %

Data in parentheses represent 95 % confidence intervals. ^a^ Suspicious scans deemed positive, indeterminate scans deemed negative. ^b^ Excludes suspicious and indeterminate scans. MR – magnetic resonance, PPV – positive predictive value, NPV – negative predictive value, LR + – positive likelihood ratio, LR - – negative likelihood ratio

### 3.2 Microbiologic results

Microbiologic results from 75 episodes in which microbiologic specimens were sent from bone for culture are shown in Table 4. Most of the bone specimens yielded pathogens typically found in diabetes-related foot infections, regardless of the histologic presence of osteomyelitis. Of the two patients with false-negative bone cultures, both were on antimicrobial therapy at the time of the biopsy.

**Table 4:** Bone microbiology of those in whom histology and bone microbiology was performed ( N= 75).

	Positive	Negative
	histology	histology
Positive culture	39	23
*S. aureus*	22	12
*S. aureus* (methicillin-resistant)	12	8
Group B streptococci	11	2
Enterococcus species	9	6
Gram-negative aerobic rods	15	12
Anaerobic bacteria	13	5
Negative culture	2	9

### 3.3 Outcomes/need for amputation

Overall, an amputation was performed in 54 of 84 episodes within 1 year of the MR scan (Table 5). Most of these were toe or metatarsal amputations, but 4 were below the knee. Among the 15 episodes with diagnostic biopsies and positive histology, an amputation was performed in 5, including 1 below the knee. Neither the presence of an abnormal MR scan nor the presence of histologic evidence of osteomyelitis correlated with the performance of a subsequent amputation.

**Table 5:** Outcomes by amputation at 1 year.

	No	Toe or	Below-knee
	amputation	metatarsal amputation	amputation
Overall	30	50	4
Histology +	11	35	4
Histology -	19	15	0
Diagnostic biopsy performed MR ${+}^{a}$	20	10	1
Diagnostic biopsy performed and MR ${-}^{b}$	9	3	0
Diagnostic biopsy performed histology +	10	4	1
Diagnostic biopsy performed histology -	19	9	0

^a^ Includes suspicious MR scans. ^b^ includes indeterminate MR scans.

## 4 Discussion

The recently published International Working Group on the Diabetic Foot/Infectious Diseases Society guidelines recommend performance of a MR scan when the diagnosis of DFO remains in doubt despite clinical, plain film radiographs and laboratory findings (Senneville et al., 2023). In previously published meta-analyses, the sensitivity of MR scanning was 93 %–96 % and specificity 75 %–82 % (Lauri et al., 2017; Llewellyn et al., 2020). Most studies defined osteomyelitis based on clinical and radiographic criteria and included patients without histologic confirmation. Only limited small studies that have evaluated the performance of MR scanning in osteomyelitis have analyzed results of MR scanning in patients in which the diagnosis of osteomyelitis was confirmed by histology (Enderle et al., 1999; Ertugrul et al., 2006). In those studies, only five patients were included that had a negative histology. In another report and review in which cases of osteomyelitis were included if either the biopsy histology *or* culture was positive, the specificity was 34 % based on the official initial radiographic read in which indeterminate results were considered negative (La Fontaine et al., 2021). When utilizing a second read only by musculoskeletal trained radiologists, the specificity improved to 74 %. It is likely that studies that included patients having osteomyelitis based on bone culture or histology included many patients with bone cultures that were positive but histology samples that were negative (Senneville et al., 2009; Lavery et al., 2019).

In our study, the sensitivity of MR scanning was high in both the group of those cases in which an amputation was performed and those with a diagnostic biopsy. Specificity was poor. Among the patients in which a diagnostic biopsy was performed who had a disease prevalence of 34.9 %, the positive likelihood ratio was only 1.75 and resultant positive predictive value only 48.4 %. Therefore, a negative test was very helpful in excluding the presence of osteomyelitis; however a positive test was of very limited diagnostic value. These results are similar to a study regarding the utility of MR imaging in the diagnosis of osteomyelitis beneath pressure sores in patients with spinal cord injuries, where the sensitivity was 94 % but specificity only 22 % in histologically confirmed cases (Brunel et al., 2016). The optimal radiographic test with a high specificity in the diagnosis of osteomyelitis is uncertain. Modalities with promise could include 18F-FDG PET (Jin et al., 2024; Lauri et al., 2017), Tc-99m HMPAO WBC scanning (Lauri et al., 2017), dynamic contrast-enhance MRI (Diez et al., 2020), or diffusion-weighted MRI (Basu et al., 2007) but have not been evaluated in patients with histologic confirmation of disease. Probe-to-bone test or the presence of exposed bone likely has higher specificity than MR imaging (Dinh et al., 2008). Although diagnostic bone biopsy has not been well evaluated prospectively to determine if its use improves outcomes, we believe it has a role to determine the presence of osteomyelitis based on histology when there is diagnostic uncertainty, as radiographic tests studied to date do not have adequate specificity to rule in disease. Bone biopsy for determination of the microbiologic etiology of the infection also enables appropriate antimicrobial therapy (Li et al., 2021).

The results of microbiology from the bone specimens (Table 4) are similar to what we (Bamberger et al., 1987) and others (Li et al., 2021) have previously reported among patients with diabetes-related foot infections. Our specimens were obtained either operatively or by use of a biopsy that traversed the wound after external cleaning of the wound with saline. The bone specimens therefore may have been contaminated by organisms in the wound, leading to the large number of positive cultures from bone specimens in which the histology was negative.

We previously reported that antimicrobial therapy without amputation can be successful in most cases of diabetes-related foot osteomyelitis, particularly in the absence of tissue ischemia (Bamberger et al., 1987). Since our study, 18 studies combined in a meta-analysis by Truong et al. (2022) have confirmed our initial findings. In the meta-analysis there was with an overall success rate of medical therapy of 68.2 %. Histologic evidence of osteomyelitis was not required as a diagnostic inclusion criterion in any of the 18 studies. The data from our current study among a limited number of patients who had a diagnostic biopsy with histologic confirmation of osteomyelitis demonstrate that 10 of 15 patients resolved their infection at 1 year without the need for an amputation and that only 1 patient required a below-knee amputation. This evidence helps confirm the role of medical therapy in the treatment of histologically confirmed diabetes-related foot osteomyelitis. Surgical therapy is likely more appropriate in the presence of concomitant uncorrected tissue ischemia, abscess, or necrotizing soft-tissue infections (Senneville et al., 2024).

In our cohort of patients in whom a diagnostic biopsy was performed, the presence of histologically confirmed osteomyelitis did not correlate with the need for amputation. Previous studies including patients in which osteomyelitis was not confirmed histologically have been conflicting, and another study including patients in which osteomyelitis was confirmed histologically did not suggest the presence of osteomyelitis was associated with amputation (Peterson et al., 1989; Mutluoglu et al., 2013; Aragón-Sánchez et al., 2008). We believe that the driving factors in the need for amputation are likely more related to the presence of vascular disease and the extent of the wound rather than the presence of osteomyelitis.

The strengths of our study include its size, the follow-up on the encounters, the use of histologic confirmation, and a second read of the MR images by a musculoskeletal radiologist using guideline criteria. The use of histology as the standard of diagnosis is the most conservative methodology (Lavery et al., 2019). Our podiatrists obtained their diagnostic biopsies using a large needle but typically traversed the wound. This would have likely diminished the chance of sampling error in obtaining the histologic sample, but the chance for sampling error is still feasible and likely would have increased the possibility of the microbiologic sample being contaminated with surface organisms from the wound compared to a transcutaneous biopsy. The weaknesses of our study include that it was retrospective from a single site, that histologic reads from the pathologists may not always be consistent, and that MR reads even with a second read can be subjective (La Fontaine et al., 2021; Lavery et al., 2019). There was a high disease prevalence of osteomyelitis in our study, and most encounters were in hospitalized patients with surrounding soft tissue infection. Patients may not have been included in the study if negative MR scans would have led to a decision not to proceed with biopsies or if a patient in which osteomyelitis was deemed highly likely based on plain radiographs, probe-to-bone testing, and/or exposed bone may not have had an MR scan to confirm disease, which may have skewed our sample group.

## 5 Conclusion

In conclusion, we found that the specificity, positive likelihood ratio, and positive predictive value of MR scanning were inadequate to rule in a diagnosis of histologically confirmed osteomyelitis, as a positive test had a low predictive value and a likelihood ratio below 2. MR scanning was helpful if negative to rule out disease if pretest probability was moderate. We provided additional evidence that antimicrobial therapy without surgical resection can be successful in the management of diabetes-related foot osteomyelitis, even in histologically proven disease.

## Data Availability

De-identified patient data can be provided upon reasonable request from the corresponding author. Data are located in a controlled access data storage at the University of Missouri-Kansas City.
